# Halogenated Phenolic Ingredients of Household and Personal Care Products Modulate Thyroid Receptor Signaling

**DOI:** 10.1021/acsomega.6c00246

**Published:** 2026-03-17

**Authors:** Veronika Weiss, Nuša Jud, Martina Gobec, Žiga Jakopin

**Affiliations:** † Department of Pharmaceutical Chemistry, 63721 Faculty of Pharmacy, University of Ljubljana, Ljubljana SI-1000, Slovenia; ‡ Department of Clinical Biochemistry, Faculty of Pharmacy, University of Ljubljana, Ljubljana SI-1000, Slovenia

## Abstract

Endocrine-disrupting chemicals can interfere with thyroid hormone signaling and may contribute to adverse health outcomes. Among these, halogenated phenolic derivatives of household and personal care product (HPCP) ingredients are of increasing concern due to their structural resemblance to thyroid hormones. In this study, a comprehensive library of halogenated parabens, bisphenols, UV filters, and nonylphenols was evaluated for thyroid receptor (TR) modulatory activity using a GH3.TRE-Luc reporter assay. Halogenated bisphenol F derivatives displayed pronounced TR agonistic activity, with dichlorinated BPF emerging as the most potent agonist. In contrast, dihalogenated long-chain parabens, particularly dibrominated analogs, demonstrated antagonistic effects in the low micromolar range. Overall, our findings demonstrate that halogenation significantly influences TR modulation by phenolic HPCP ingredients, emphasizing the need for further investigation into their potential endocrine-disrupting impact.

## Graphical abstract

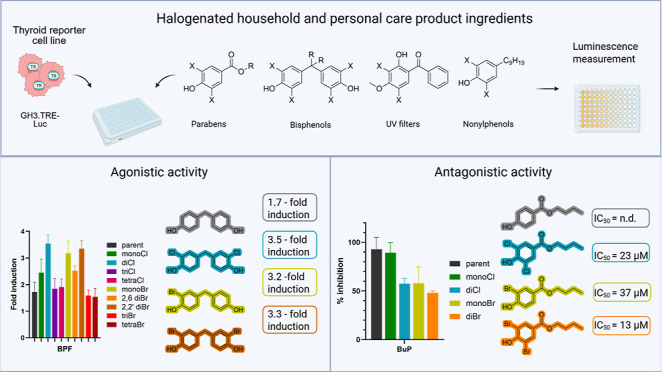

## 1 Introduction

Numerous natural and synthetic endocrine-disrupting chemicals (EDCs) adversely affect the endocrine system, including the thyroid axis.^1^ Thyroid function is governed by the hypothalamus (releasing thyrotropin-releasing hormone), the pituitary gland (releasing thyroid-stimulating hormone), and the thyroid, which produces the hormones triiodothyronine (T3) and thyroxine (T4). These play crucial roles in growth, development, and metabolic homeostasis,^2^ acting through thyroid hormone receptors (TRs) which are ligand-dependent transcription factors that bind DNA response elements to regulate gene expression.^3^ Given its central role in development, especially brain maturation in fetuses and children, thyroid dysfunction can have severe consequences, including cognitive impairment, metabolic disorders, and cancer.^4^

Humans are exposed to various industrial and consumer product chemicals that interfere with thyroid signaling at multiple prereceptor levels, such as hormone synthesis, secretion, transport (e.g., transthyretin), metabolism (e.g., glucuronidation, sulfation, deiodination), and cellular uptake.^5^ However, many EDCs also act directly on TRs as agonists or antagonists, or modulate TR expression.^6^ Environmental pollutants such as polychlorinated biphenyls (PCBs), polybrominated diphenyl ethers (PBDEs), and their hydroxylated metabolites that structurally mimic thyroid hormones and disrupt homeostasis by competitively binding to TRs.^7,8^ Hydroxylated PCBs and PBDEs also bind transthyretin and affect thyroid metabolic enzymes. Other chemicals, including perchlorates, pesticides, bisphenols, nonylphenols, phthalates, parabens, and UV filters, have demonstrated thyroid activity.^9^

Parabens, bisphenols, certain chemical classes of UV filters, and nonylphenol share a phenolic moiety, making them prone to halogenation reactions that occur in environments such as wastewater treatment plants or swimming pools exposed to sodium hypochlorite or brominated disinfectants.^10^ Resulting chlorinated and brominated transformation products have been detected in the environment (e.g., chlorinated parabens up to 4400 ng/L) and in humans (e.g., chlorinated bisphenols in urine up to 1500 ng/L).^9^ Structurally, these halogenated phenolics resemble thyroid hormones; the combination of a hydroxyl group adjacent to a halogen atom is known to confer TR binding affinity.^11,12^ In T3, the hydroxyl forms a hydrogen bond with TR, while only one iodine atom interacts directly with the receptor. The remaining iodine atoms contribute to structural rigidity, enforcing noncoplanarity of the two aromatic rings, and increasing molecular bulk.^13^ While it has been established that halogenated phenolic compounds in general have the potential to bind TR, little is known about the thyroid receptor activity of chlorinated and brominated parabens, bisphenols, UV filters, and nonylphenols.^14,15^ Only two prior studies reported TR activity for specific members of this group, showing that chlorinated bisphenol A and chlorinated nonylphenol exhibit stronger TR binding than their nonhalogenated counterparts.^16,17^

To address this gap, we investigated a comprehensive library of halogenated transformation products derived from phenolic ingredients in household and personal care products (HPCPs)^18^ and screened them for thyroid receptor–modulating activity using the GH3.TRE-Luc reporter cell line. Both agonistic and antagonistic effects were evaluated at two concentrations (1 and 10 μM), alongside assessment of metabolic activity. Noncytotoxic compounds showing significant TR modulation in preliminary screening underwent further evaluation for dose dependence.

## 2 Results and Discussion

Altogether, a chemical library encompassing 125 compounds was screened for thyroid receptor modulatory activity. The compounds were initially examined for their effect on metabolic activity using the CellTiter 96 Aqueous One Solution cell proliferation assay to distinguish receptor-mediated effects from potential cytotoxicity-related artifacts. Compounds that exhibited notable activity on the thyroid receptor at noncytotoxic concentrations, either in an agonistic or antagonistic manner, were subsequently evaluated in dose–response experiments to further characterize their activity profiles.

### 2.1 Metabolic Activity

To assess the metabolic activities, GH3.TRE-Luc cells were treated for 24 h with all 125 compounds from the chemical library, including the unsubstituted, chlorinated and brominated derivatives of phenolic HPCP ingredients at two different concentrations (1 and 10 μM). The metabolic activity was tested in an agonistic (presented in Figure S1) as well as in an antagonistic (presented in Figure S2) setup, the latter involving cotreatment with T3 (final concentration 0.25 nM). The metabolic activity data also served as an important control to distinguish receptor-mediated effects from potential cytotoxicity-related artifacts.

Exposure of GH3.TRE-Luc cells to the tested compounds revealed that most parent parabens did not substantially affect metabolic activity, whereas several halogenated derivatives, particularly dibrominated parabens, produced moderate reductions at higher concentrations (e.g., Br_2_iPrP (83%), Br_2_BuP (84%) and Br_2_PeP (75%)). A notable exception was seen for Cl_2_BzP and Br_2_BzP, which caused marked decreases of metabolic activity to 55% and 45%, respectively. Of the tested bisphenols, a few caused a minor drop in metabolic activity by reducing it to 75–85% at the highest tested concentration of 10 μM, which was in agreement with the report of Ghisari et al., wherein 20 μM BPA was devoid of cytotoxicity on GH-3.^19^ Conversely, our study revealed that a series of halogenated BPAF derivatives had more pronounced effects on the metabolic activities decreasing them to 38–76% (Figure S1). These observations indicate cytotoxic activity of the halogenated BPAF derivatives, in particular at 10 μM. An interesting phenomenon was observed in cells treated with various UV chemotypes. In particular, the metabolic activity was unexpectedly elevated to approximately 150% in all halogenated benzophenone-3 (BP-3) derivatives. On the other hand, among the dibenzoylmethanes a marked drop was seen with the dichlorinated BMDM (avobenzone) analog (31%), which could also be attributed to the cytotoxicity.

Interestingly, the metabolic activities of cells treated with parabens and representatives of all three tested UV filter chemotypes (benzophenones, dibenzoylmethanes, and cinnamates) in an antagonistic setup were also elevated to approximately 140–210%, as shown in Figure S2A,B. Similar effects were observed with bisphenols, albeit to a lesser extent. This could be ascribed to the presence of T3, which increased the metabolic activity of the cells by itself (compared to DMSO). This phenomenon aligns with the findings from Ghisari et al., where T3 notably induced proliferation of GH3 cells, while the tested endocrine disruptors exhibited minor effects on the proliferation increase.^19^ Nonetheless, similar to what has been observed in the agonistic setup, treatment with Cl_2_BMDM at 10 μM concentration also decreased the metabolic activity. It is worth mentioning that none of the compounds decreased the metabolic activities in either agonistic or antagonistic setup at the 1 μM concentration.

### 2.2 TR Agonistic Activity Screening

The thyroid receptor agonistic activity screening results of our chemical library of parent and halogenated transformation products obtained for two different concentrations, 10 and 1 μM, are presented in Figures 1 and S3, respectively. Briefly, the compounds’ agonistic activities were determined employing the GH3.TRE.Luc reporter cell line by measuring luciferase secretion, which is directly correlated to compound-induced activation of TR. The natural TR agonist, 3,3′,5-triiodo-l-thyronine (T3, 100 nM), increased receptor activity 5.6-fold compared to DMSO. Screening of agonistic activity demonstrated that halogenation differentially affected TR activation depending on the phenolic scaffold. Among parabens, parent compounds possessing longer alkyl side chains (iBuP, PeP, and BzP) showed modest agonistic activity (1.5–1.7-fold) at 10 μM, whereas halogenated paraben derivatives were largely inactive as TR agonists. These results are in partial agreement with Liang et al., who observed TR-mediated proliferation of GH3 cells exposed to MeP, EtP, PrP, and BuP, though only at much higher concentrations (50–500 μM).^20^ Similarly, Taxvig et al. found EtP inactive in a T-screen assay, whereas BuP acted as a TR agonist at 10 nM–30 μM, enhancing proliferation up to 300%.^21^ The ability of BuP to further stimulate proliferation in the presence of T3 was also consistent with our findings. A study examining maternal exposure to parabens revealed that exposure to MeP during early pregnancy significantly increased TSH levels in female twins.^22^ Also, Coiffier et al. observed elevated TSH levels in both boys and girls exposed to BuP.^23^ Conversely, a study of Berger et al. reported that exposure to MeP and PrP during pregnancy resulted in lower TSH levels in women.^24^ It should be noted that our in vitro results cannot directly be translated and compared to those obtained in in vivo studies, due to vast differences in the complexity level, such as binding to transport proteins, disrupting metabolism, etc.

**Figure 1:**
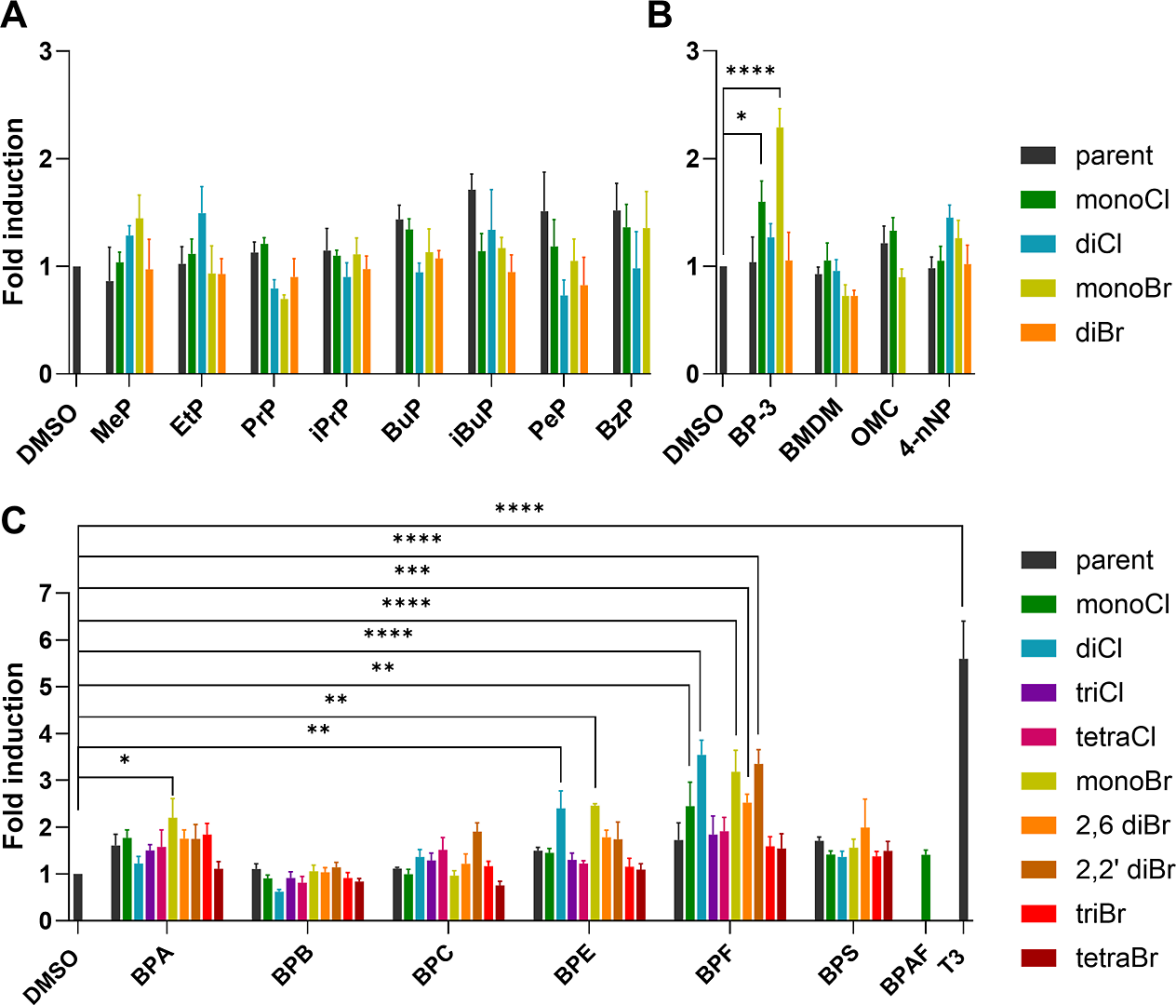
Relative thyroid receptor agonistic activity of compounds at 10 μM concentration. GH3.TRE.Luc cells were treated with (A) parabens, (B) UV filters and nonylphenols, and (C) bisphenols. DMSO was used as the negative control (0.1%) and T3 was used as the positive control (100 nM). After 24 h, luminescence was measured. Results are presented as means ± SEM of four independent experiments. Statistical significance between tested compounds versus negative control (DMSO) was calculated using one-way ANOVA post hoc Dunnett’s test. (****p < 0.0001; ***p < 0.001; **p < 0.01; *p < 0.05; NS not significant). The bars for cytotoxic compounds are not shown in the Figure.

In contrast, several halogenated bisphenol derivatives exhibited pronounced TR agonistic activity indicating that moderate halogenation enhances agonistic potential within this chemical class. For example, a number of halogenated BPE derivatives showed moderate TR agonistic activity, including Cl_2_BPE (2.4-fold), BrBPE (2.5-fold), 2,6-Br_2_BPE (1.8-fold), and 2,2′-Br_2_BPE (1.7-fold). Most halogenated BPA derivatives produced less than a 2-fold increase, while only 2,2′-Br_2_BPC (1.9-fold) and 2,6-Br_2_BPS (2.0-fold) were active among BPB, BPC, and BPS analogs. Except for ClBPAF, all BPAF derivatives reduced metabolic activity at 10 μM and showed no agonism at 1 μM (Figure S3). Notably, mono- and dihalogenated BPF derivatives induced TR activity above 3-fold, prompting further dose–response assessment. These results partly agree with Zhang et al., who reported GH3 cell proliferation by BPA, BPF, and BPS at 10–50 μM, with BPA showing the highest (2.7-fold) effect.^25^ BPA and BPF also activated TR in our study, though concentrations above 10 μM were excluded due to cytotoxicity. Additionally, Kitamura et al. reported that TBBPA and TCBPA increased growth hormone release from GH3 cells at concentrations of 10 and 100 μM, suggesting their agonistic activity.^26^ Our results partially corroborate these findings, showing a slight increase in TR agonistic activity for TCBPA but no changes in cells treated with TBBPA. Hofmann et al. also demonstrated that TBBPA acted as a TR agonist at 10 μM in a reporter assay using transfected HepG2 cells, further supporting the potential receptor-modulating properties of certain halogenated bisphenols.^27^

Among UV filters, only benzophenone BrBP-3 induced moderate receptor activation (2.3-fold), while representatives of other tested UV filter chemical classes and their halogenated analogs were inactive. Similarly, Hofmann et al. reported modest TR activation by BP-3 and OMC (1.8- and 1.5-fold) in HepG2 cells.^27^ Nonylphenol and its halogenated derivatives also showed no TR agonistic activity, consistent with results of Ji et al. obtained in yeast-two hybrid assay.^28^ However, Schmutzler et al. found NP to act as a TR agonist in HepG2 cells,^29^ and Wang et al. demonstrated in vivo alterations in thyroid hormones and histopathology in NP-treated rats, suggesting potential thyroid-disrupting effects under physiological conditions.^30^

Only compounds showing the strongest TR agonistic effects at noncytotoxic concentration in the initial screening were evaluated for dose dependence. Cl_2_BPF, BrBPF, and 2,2′-Br_2_BPF increased TR activity more than 3-fold, prompting further testing of all halogenated BPF derivatives to assess the impact of halogenation degree on TR activation. As shown in Figure 2, dose–response analysis confirmed the screening results: ClBPF (2.3-fold) and Cl_2_BPF (2.5-fold at 25 μM) were more potent agonists than parent BPF. In contrast, Cl_3_BPF showed a minor increase at higher concentrations, while Cl_4_BPF was inactive. A similar trend was observed for brominated analogs, BrBPF and 2,2′-Br_2_BPF enhanced TR activity (2.7- and 2.3-fold at 25 μM), whereas Br_3_BPF and Br_4_BPF were inactive. EC_50_ values could not be determined, as no compound reached a response plateau due to decreased metabolic activity at the highest concentration; thus, fold inductions at 25 μM were reported (Table S1).

**Figure 2:**
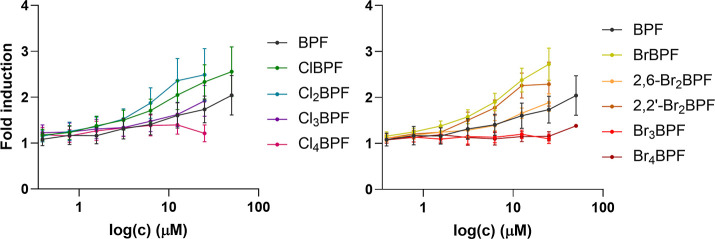
Dose–response curves from four biological repeats (done in duplicates). All results are presented as means ± SEM.

### 2.3 TR Antagonistic Activity Screening

The TR antagonistic activity screening results of the halogenated transformation products and their parent compounds obtained in the luciferase reporter assay at both 10 μM and 1 μM are presented in Figures 3 and S4, respectively. Briefly, the GH3.TRE.Luc cells were treated with all compounds and the positive control NH-3 (TR antagonist), and then stimulated with the bona fide TR agonist T3 (final concentration 0.25 nM). The commercially available antagonist NH-3 at 100 nM decreased the T3-induced TR activity (i.e., residual activity) to 34%. Unsubstituted parabens MeP, EtP, PrP, and iPrP as well as their halogenated derivatives showed no significant antagonism, while parent BuP, PeP, and BzP were also inactive; notably, iBuP slightly enhanced TR activity, acting additively with T3. In contrast, Liang et al. reported that MeP (20 μM), EtP (20 μM), PrP (5 μM) and BuP (5 μM) decreased levels of T3 and T4 in zebrafish larvae, however zebrafish is a fundamentally different model compared to our cell line and the underlying mechanism may differ.^20^ While, our in vitro model affords very specific information, direct activation or inhibition of TR mediated transcription, the zebrafish model can reveal multilevel thyroid disruption, such as inhibition of hormone synthesis, disruption of hormone transport, metabolism or clearance. Notably, parent parabens demonstrated agonistic behavior as described in chapter 2.2. On the other hand, the halogenated derivatives of parabens incorporating longer side chains (BuP, iBuP, PeP and BzP) showed more pronounced antagonistic activities dependent on the degree of halogenation (as shown in Figure 3).

**Figure 3:**
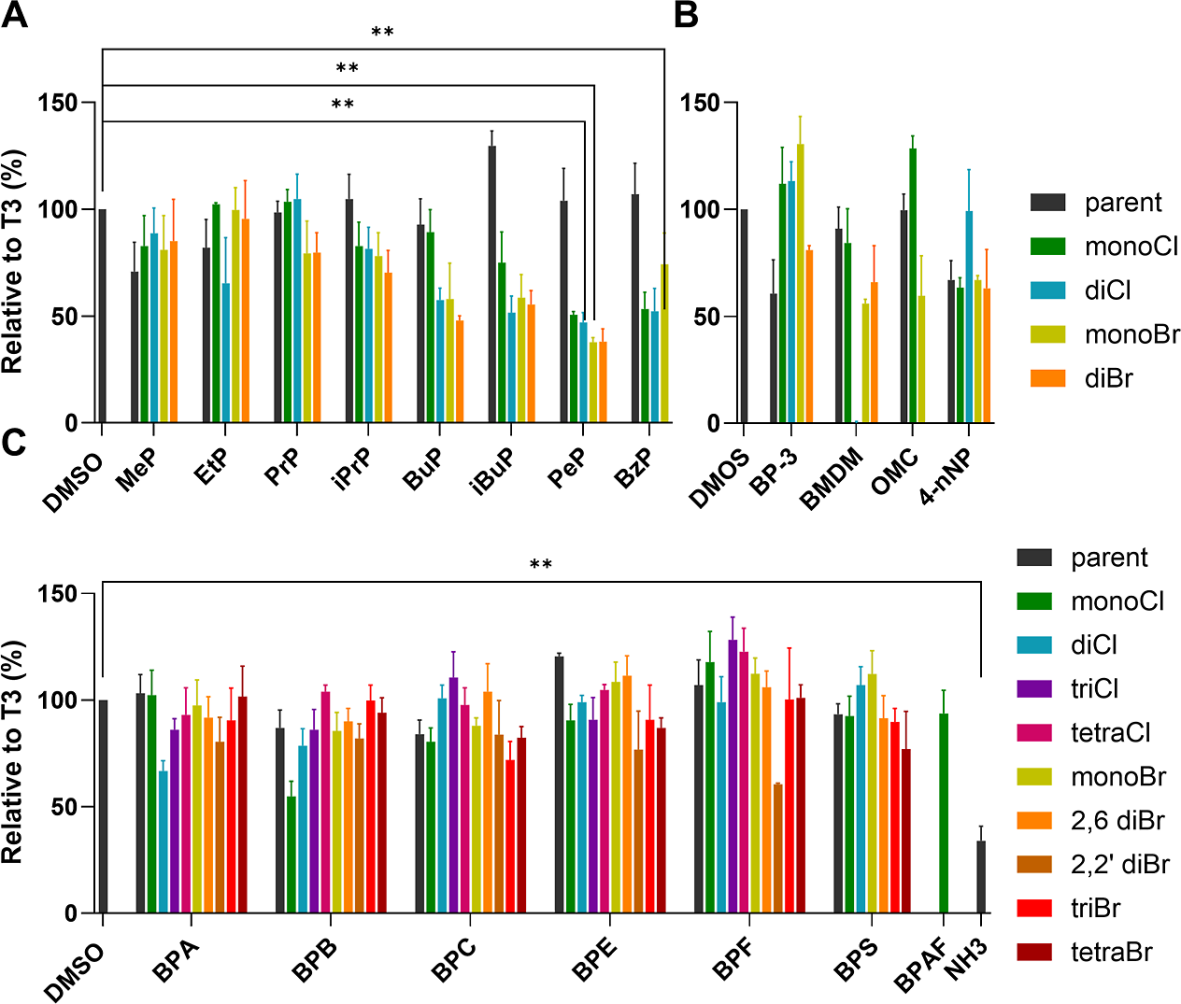
Relative thyroid receptor antagonistic activity of compounds at 10 μM concentration. GH3.TRE.Luc cells were treated with (A) parabens, (B) UV filters and nonylphenols and (C) bisphenols. After 1h, 0.25 nM of T3 was added to each well. DMSO was used as the negative control (0.1%) and NH-3 was used as the positive control (100 nM). After 24 h, luminescence was measured. Results are presented as mean ± SEM of four independent experiments. Statistical significance between tested compounds versus negative control (DMSO) was calculated using one-way ANOVA post hoc Dunnett’s test (**p < 0.01; *p < 0.05). The bars for cytotoxic compounds are not shown in the Figure.

Specifically, BuP, ClBuP, Cl_2_BuP, BrBuP and Br_2_BuP at 10 μM inhibited the T3-elicited activity to 92%, 89%, 57%, 58% and 48%, respectively. Similarly, their halogenated PeP counterparts (PeP, ClPeP, Cl_2_PeP, BrPeP and Br_2_PeP) carrying a C5 side chain, decreased the activity to 104%, 50%, 47%, 38% and 38%, respectively. These findings suggest that antagonistic potency of the compounds belonging to this particular chemotype increases with increasing carbon side chain length as well as with increasing degree of halogenation. Clearly, the antagonistic activity of the dihalogenated derivatives (i.e., Cl_2_BuP, Br_2_BuP, Cl_2_PeP, Br_2_PeP) was more pronounced compared to their monohalogenated counterparts. In addition, the brominated analogs proved to be more potent antagonists than their chlorinated congeners.

Among bisphenols, antagonistic activity was less pronounced. None of the parent bisphenols or their halogenated derivatives showed TR antagonistic activity at 1 μM, while only Cl_2_BPA, ClBPB, and 2,2′-Br_2_BPF at 10 μM reduced T3-induced activity to 67%, 55%, and 61%, respectively. All halogenated BPAF derivatives also decreased metabolic activity at this concentration. Our results partially align with study of Kitamura et al., who found no TR effects for BPA, BPB, BPF, BPS, or BPAF.^26^ In contrast, Moriyama et al. reported BPA antagonism toward TRα and TRβ at 1–100 μM in TSA201 cells,^31^ and Collet et al. observed BPA and TBBPA acting as TRβ antagonists (IC_50_ = 11 μM and 0.85 μM).^32^ Additionally, Lee et al. showed that exposure of GH3 cells to 10 mg/L of BPA (approximately 44 μM) significantly downregulated *tr*α and *tr*β genes.^33^ Interestingly, BPF significantly suppressed the transcription of those genes at concentrations one or 2 orders of magnitude lower than that of BPA, which is contrary to our results, which indicate that BPF acts as an agonist. The same study reported that BPS downregulated *tr*α and *tr*β genes at midmicromolar range, a finding not observed in our experiments.

Similarly, several derivatives of various UV filter chemotypes as well as nonylphenol derivatives exhibited modest or inconsistent antagonistic effects. The reduction was observed in parent BP-3, both brominated BMDM, BrOMC, and nonylphenols with the exception of Cl_2_NP. Similarly, Lee et al. reported that BP-3 significantly downregulated *tr*β, *tsh*β, and *trhr* gene expression in GH3 cells.^34^ In zebrafish larvae, BP-3 caused a significant decrease in T3 levels without affecting T4 levels at a concentration of 32 μg/L (approximately 0.14 μM). Comparable effects were observed for BMDM and OMC, which reduced T3 and T4 levels in wild-type zebrafish larvae at 30 μM.^35^ Additionally, rats administered 12.5 g/kg of OMC exhibited lower total T4 concentrations, although T3 levels remained unaffected.^29^ Collet et al. reported NP antagonism toward TRβ in yeast and mammalian cell assays (IC_50_ = 3.5 μM),^32^ and Schmutzler et al. observed elevated T3 and T4 levels in rats treated with 80 mg/kg 4-NP.^29^ Our observations are only partially in line with other studies, since we have not observed any effect in OMC-treated cells, however *ex vitro* and in vivo results cannot be directly compared to results from in vitro assays.

The dose dependence studies were than carried out for the noncytotoxic compounds exhibiting the most pronounced antagonistic activities in the general screening, namely the halogenated derivatives of BuP and PeP (the cut off value was set at 50% of residual response; the corresponding parent compounds were included for comparison). In Table 1, determined potencies of selected compounds and their induced responses at 25 μM are listed; their dose–response curves are shown in Figure 4. Simultaneously, metabolic activity was assessed in order to determine nontoxic concentrations (data not shown). Since a decrease in metabolic activity emerged at high concentrations (25 or 50 μM, depending on the compound), the bottom values were constrained to the lowest point reached by positive control −NH-3 (13%).

**Table 1:** Approximate IC_50_ Values of the Tested Compounds

compound	IC_50_ (μM)	residual activity at 25 μM (%)
BuP	n.d	130 ± 4.94
ClBuP	47 ± 4.3	98.5 ± 4.81
Cl_2_BuP	23 ± 3.2	53.8 ± 2.03
BrBuP	37 ± 3.4	83.1 ± 2.40
Br_2_BuP	13 ± 1.4	27.5 ± 0.955
PeP	54 ± 11	125 ± 7.76
ClPeP	22 ± 1.8	54.0 ± 0.471
Cl_2_PeP	17 ± 4.7	43.0 ± 0.471
BrPeP	21 ± 1.1	48.3 ± 2.42
Br_2_PeP	9.5 ± 1.5	49.0 ± 2.16^a^
NH-3	85 nM ± 21 nM	15.9 ± 1,47^b^

a Calculated for inhibition at 12.5 μM due cytotoxicity at 25 μM.

b Calculated inhibition at 1 μM.

**Figure 4:**
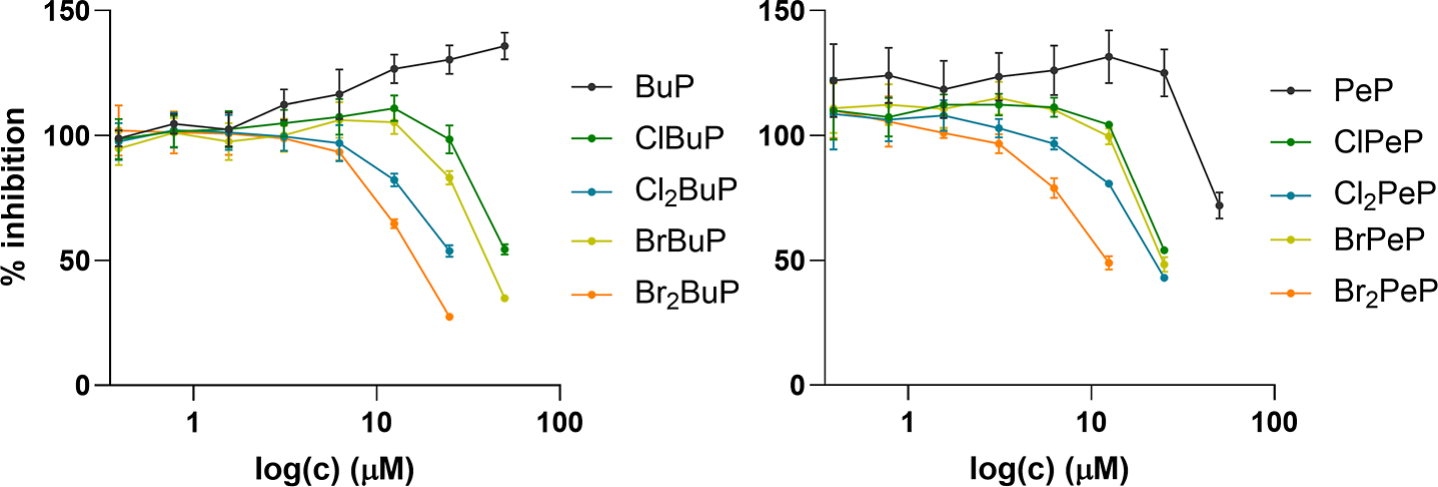
IC_50_ curves from at least three biological repeats (done in duplicates). All results are presented as means ± SEM.

Albeit the IC_50_ values listed in Table 1 are approximate values, still some trends can be distinguished. The parent BuP exhibited no antagonistic effect, while its homologue PeP decreased the T3-induced activity at 50 μM. Consistent with results from the screening assays conducted at 1 and 10 μM, halogenation enhanced the antagonistic activities of BuP and PeP. Specifically, the IC_50_ values for PeP decreased from 54 μM to 22 μM (ClPeP), 21 μM (BrPeP), 17 μM (Cl_2_PeP) and 9.5 μM (Br_2_PeP). A similar trend was observed for BuP and its mono- and dihalogenated analogs, demonstrating that dihalogenated parabens were more potent antagonists than their monohalogenated and unsubsituted counterparts. Furthermore, bromination proved more effective than chlorination in inhibiting TR activity. Due to a drop in metabolic activity, the lowest induced response could not be determined, so the extent of inhibition was compared at 25 μM. At this concentration, BuP and PeP seemingly acted additively with T3, enhancing its agonistic activity by elevating it to 130% and 125%, respectively. A comparable effect was reported in a T-screen assay using GH3 cells treated with a combination of BuP and T3.^21^ In contrast, the halogenated PeP derivatives at 25 μM significantly reduced activity, with inhibition levels of 54% (ClPeP), 43% (BrPeP), 48% (Cl_2_PeP), and 49% (Br_2_PeP at 12.5 μM). As expected, the reference antagonist NH-3 was the most potent (IC_50_ = 85 nM), lowering activity to 16% at 1 μM. Dose–response experiments thus confirmed that halogenation enhances antagonistic potency within the paraben series.

Interpretation of the observed TR-modulating activity should consider several limitations inherent to the experimental model. The GH3.TRE-Luc assay reflects integrated TR-mediated transcriptional signaling but does not allow discrimination between TR isoforms. Native GH3 cells are reported to express both TRα and TRβ, with TRβ generally considered the dominant isoform in pituitary-derived systems.^19^ Consequently, the responses observed in the present study likely represent combined TR signaling with probable TRβ bias.

It should also be noted that highly halogenated parabens, particularly brominated derivatives with longer alkyl side chains, exhibit increased lipophilicity that may influence their aqueous solubility and cellular partitioning. Although cytotoxic concentrations were excluded based on metabolic activity measurements, physicochemical properties may contribute to the apparent antagonistic profiles of highly hydrophobic derivatives. Therefore, the observed activity should be interpreted as reflecting a combination of receptor-mediated and physicochemical influences. Nonetheless, the consistent structure-dependent trends suggest specific modulation patterns rather than random cytotoxic effects.

## 3 Conclusions

This study provides new insights into the thyroid receptor modulatory properties of halogenated derivatives of HPCP ingredients. Our results demonstrate that structural modification through halogenation substantially influences TR-mediated transcriptional activity. Halogenated BPF derivatives exhibited notable TR agonism, whereas the dihalogenated long-chain parabens demonstrated apparent TR antagonistic activity in the low micromolar range, although contributions of physicochemical properties cannot be fully excluded. Importantly, the GH3.TRE-Luc reporter model employed in this study is known to predominantly reflect TRβ-mediated signaling. Therefore, the observed effects likely represent TRβ-driven responses.

Beyond direct receptor modulation, halogenated phenolic endocrine disruptors may influence thyroid hormone homeostasis through additional pathways, including interference with thyroid hormone metabolism enzymes or competition for transport proteins such as transthyretin, as previously reported for structurally related halogenated pollutants.^5,11^ Considering the widespread occurrence of HPCP-derived phenolic compounds and their transformation products, the demonstrated ability of halogenation to enhance TR-modulating properties warrants further mechanistic and in vivo investigations. The present study contributes to understanding structure-activity relationships governing TR modulation and provides a foundation for future toxicological risk assessment of emerging halogenated endocrine disruptors.

## 4 Materials and Methods

### 4.1 Reagents and Synthesis

Reagents and synthesis of library of halogenated transformation products are described in detail in our previous paper.^18^ Stock solutions were prepared in DMSO and were stored in the dark at −20 °C. 3,3′,5-Triiodo-l-thyronine was obtained from Sigma-Aldrich and NH-3 from MedChemExpress.

### 4.2 Cell Culture

GH3.TRE-Luc cells are a rat pituitary tumor cell line stably transfected with TR (kind gift from Prof. T. Murk, Wageningen University, The Netherlands), and were used to identify TR agonist and antagonist activities. Native GH3 cells are known to express both thyroid receptor isoforms (TRα and TRβ), with literature indicating a predominance of TRβ expression in pituitary-derived GH3 cells. However, the isoform composition of the stably transfected GH3.TRE-Luc reporter cells has not been characterized. Therefore, the present assay is expected to reflect combined TR-mediated transcriptional signaling, with a probable bias toward TRβ-driven responses. The cells were grown and maintained in Dulbecco’s Modified Eagle Medium/Nutrient Mixture F-12 (DMEM/F-12) (Gibco, Thermo Fisher Scientific, Waltham, MA, USA), supplemented with 10% fetal bovine serum (Gibco, Thermo Fisher Scientific, Waltham, MA, USA), 100 U/mL penicillin, and 100 μg/mL streptomycin. The test medium used was DMEM/F-12 (without phenol red) supplemented with 10 μg/mL insulin, 10 μM ethanolamine, 10 ng/mL sodium selenite, 10 μg/mL human apotransferrin, and 500 μg/mL bovine serum albumin (all Sigma-Aldrich, St. Louis, MO, USA). The cells were incubated in a humidified atmosphere at 37 °C and 5% CO_2_.

### 4.3 Metabolic Activity

The tested compounds were dissolved in DMSO and further diluted in culture medium to the desired final concentrations, such that the final DMSO concentration did not exceed 0.5%. GH3.TRE-Luc cells were seeded (4 × 10^4^ cells/well) in transparent 96-well plates in 200 μL of test medium and treated with different compounds of interest at concentrations of 1 and 10 μM for initial screening, or 50, 25, 12.5, 6.25, 3.125, 1.5625, 0.78125, and 0.390625 μM for EC_50_ and IC_50_ assays. Control cells were treated with the corresponding vehicle (0.5% DMSO). After 24 h, CellTiter 96 Aqueous One Solution cell proliferation assay (Promega, Madison, WI, U.S.A.) was used according to the manufacturer’s instructions. The experiments were run in duplicates and repeated in at least two independent biological replicates.

### 4.4 Thyroid Activity

The thyroid activity was measured using a luciferase reporter assay method as previously described.^12^ The cells were seeded at 80% confluency in culture flasks in growth medium. After 24 h, the growth medium was removed, the cells were rinsed with phosphate-buffered saline and the test medium was added. After additional 24 h, 8 × 10^4^ cells in 100 μL/well were seeded in white 96-well plates and preincubated at 37 °C for 3 h. Subsequently, 100 μL of samples were added to each well. T3 (100 nM and 0.1 nM) was used as a positive control for agonistic activity. For the antagonist screening setup, a bona fide TR antagonist NH-3 (100 nM) was used as a control and the dilution medium also contained T3 (final concentration, 0.25 nM). The cells were incubated for 24 h, then ONE-Glo (Promega) was added according to manufacturer’s instructions, and luciferase luminescence was recorded (2 s medium shaking step followed by luminescence end point measurement; no light source or emission filters) using a microplate reader (Tecan Spark).

### 4.5 Statistical Analysis

All the experiments were performed at least two times, with average values expressed as means ± standard error of mean (SEM). Statistical analyses were performed using GraphPad Prism 10 (La Jolla/CA, United States).

## Supplementary Material



## Data Availability

All data supporting the findings of this study are included within the manuscript and its Supporting Information file.

## Notes

Figures S1–S4 and Table S1 are provided (PDF)
