# Supplementary material for: Halogenated Phenolic Ingredients of Household and Personal Care Products Modulate Thyroid Receptor Signaling
Source: ACS Omega. 2026 Mar 17;11(12):19782–9. doi: 10.1021/acsomega.6c00246 (PMC13044685; doi:10.1021/acsomega.6c00246)
Supplement: Supplementary file 1 [file ao6c00246_si_001.pdf]

Supporting information

Halogenated phenolic ingredients of household and personal care products modulate thyroid receptor signaling

Veronika Weiss[†], Nuša Jud[‡], Martina Gobec[‡], Žiga Jakopin[†]*

[†] Department of Pharmaceutical Chemistry, Faculty of Pharmacy, University of Ljubljana, SI-1000 Ljubljana, Slovenia

[‡] Department of Clinical Biochemistry, Faculty of Pharmacy, University of Ljubljana, SI-1000 Ljubljana, Slovenia

***Corresponding author: Žiga Jakopin**

Department of Pharmaceutical Chemistry

Faculty of Pharmacy

University of Ljubljana

Aškerčeva 7

SI-1000 Ljubljana, Slovenia

Tel: +386 1 4769646

e-mail: ziga.jakopin@ffa.uni-lj.si

Supporting Figures

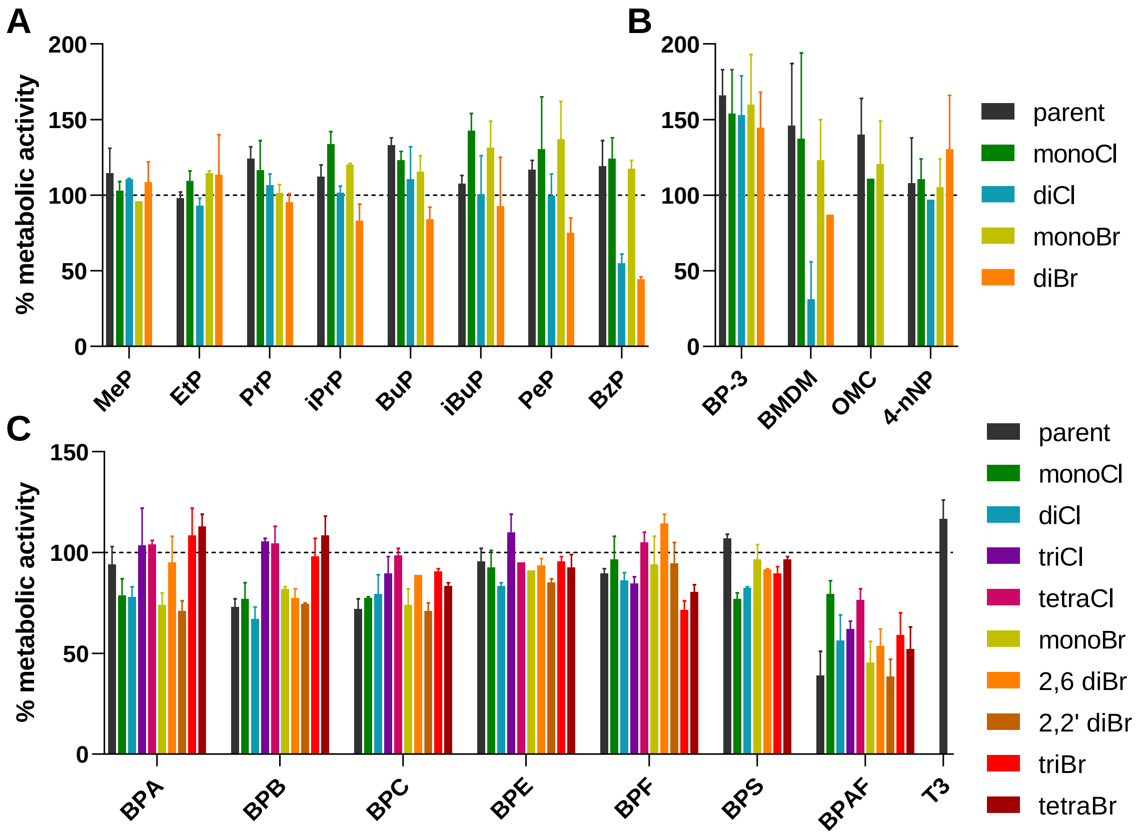

Figure S1. Metabolic activity of GH3.TRE-Luc cells after 24h treatment with 10 µM concentration of compounds – A) halogenated parabens, B) halogenated UV filters and nonylphenol derivatives, C) halogenated bisphenols – or T3 as the bona fide agonist (100 nM). The conditions used match those for the agonist activity screening. The data is presented as mean ± SEM.

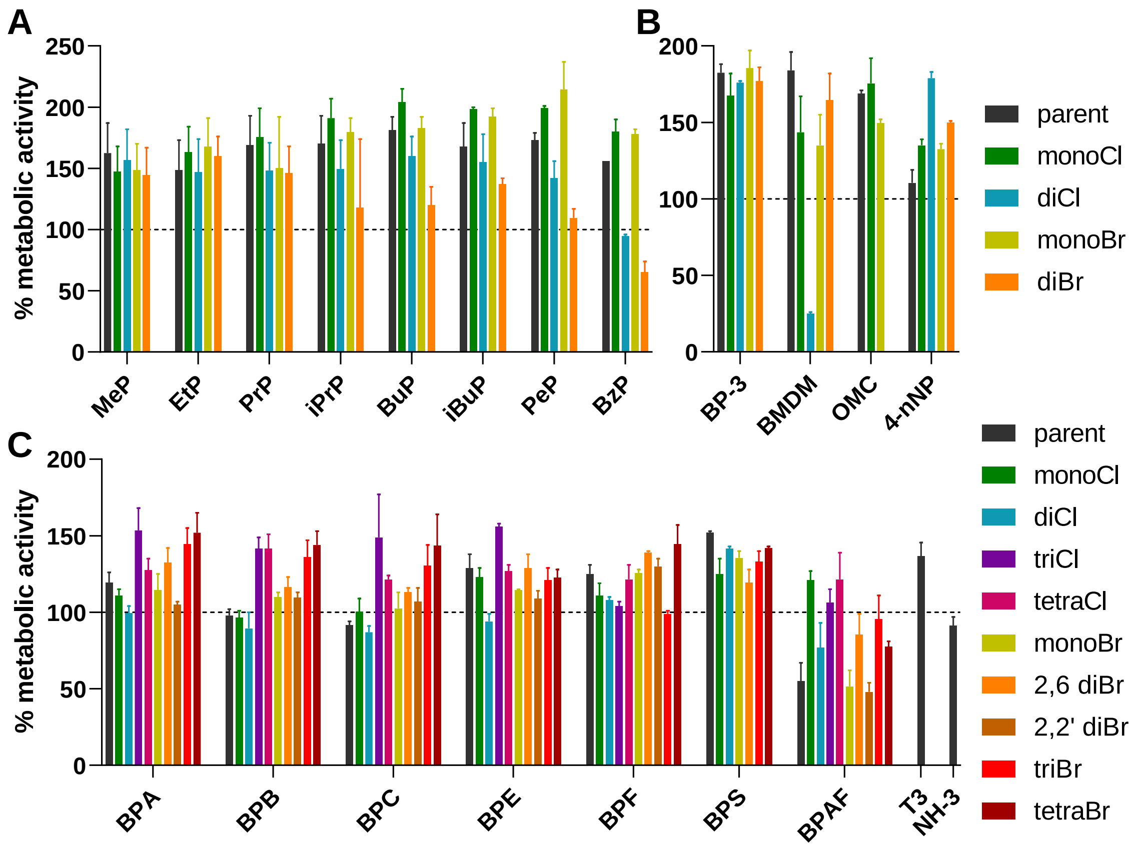

Figure S2. Metabolic activity of GH3.TRE-Luc cells after 24h co-treatment with the control T3 (0.25 nM) and 10 µM concentration of compounds – A) halogenated parabens, B) halogenated UV filters and nonylphenol derivatives, C) halogenated bisphenols – or NH-3 (100 nM) as the bona fide TR antagonist. The conditions used match those for the antagonist activity screening. The data is presented as mean ± SEM.

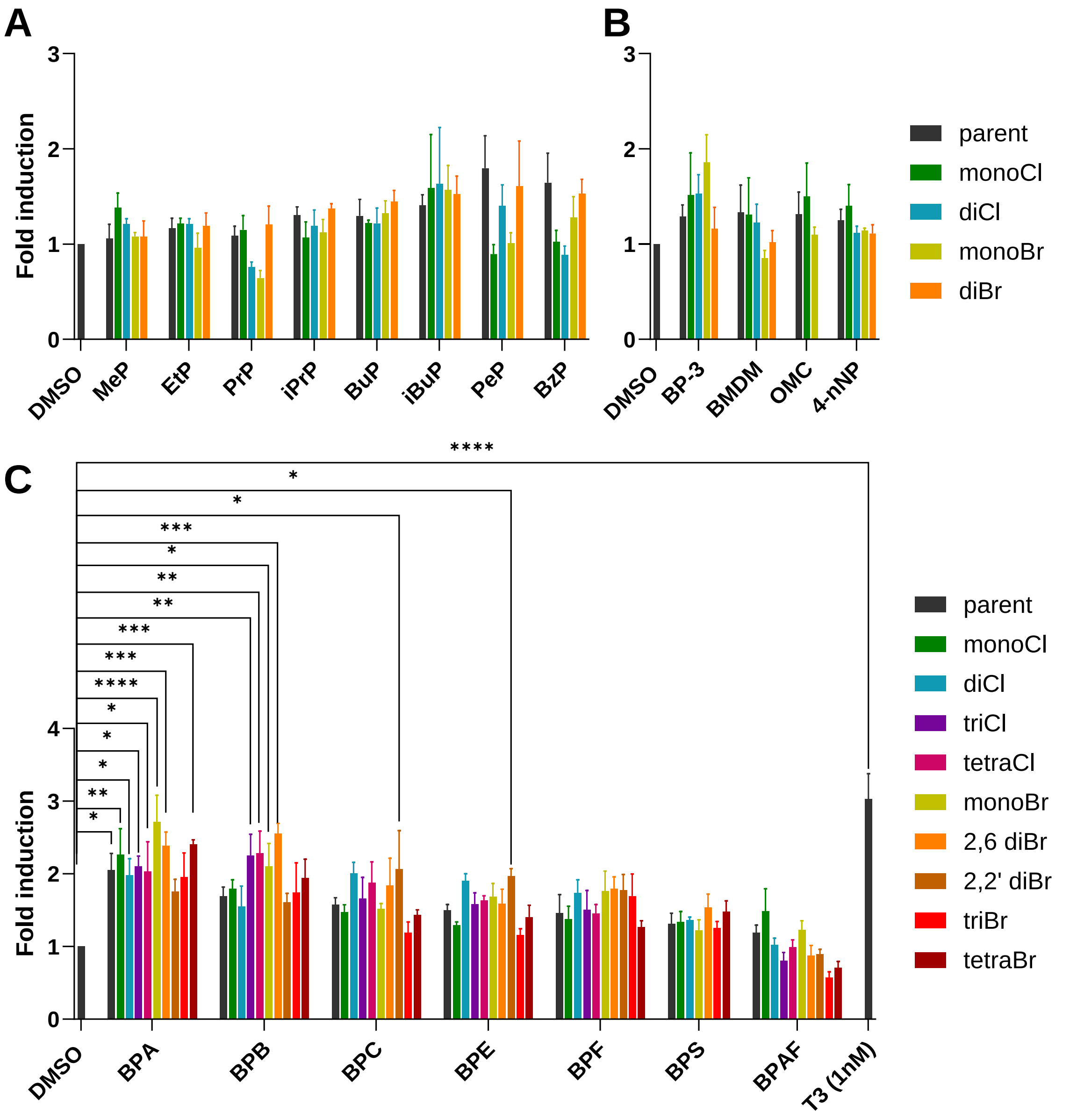

Figure S3. Relative thyroid receptor agonistic activity of compounds at 1 µM concentration. GH3.TRE.Luc cells were treated with (A) parabens, (B) UV filters and nonylphenols, and (C) bisphenols. DMSO was used as the negative control (0.1%) and T3 was used as the positive control (1 nM). After 24 h, luminescence was measured. Results are presented as means ± SEM of four independent experiments. Statistical significance between tested compounds versus negative control (DMSO) was calculated using one-way ANOVA post hoc Dunnett's test. (****$p<0.0001$; ***$p < 0.001$; **$p < 0.01$; *$p < 0.05$; NS not significant).

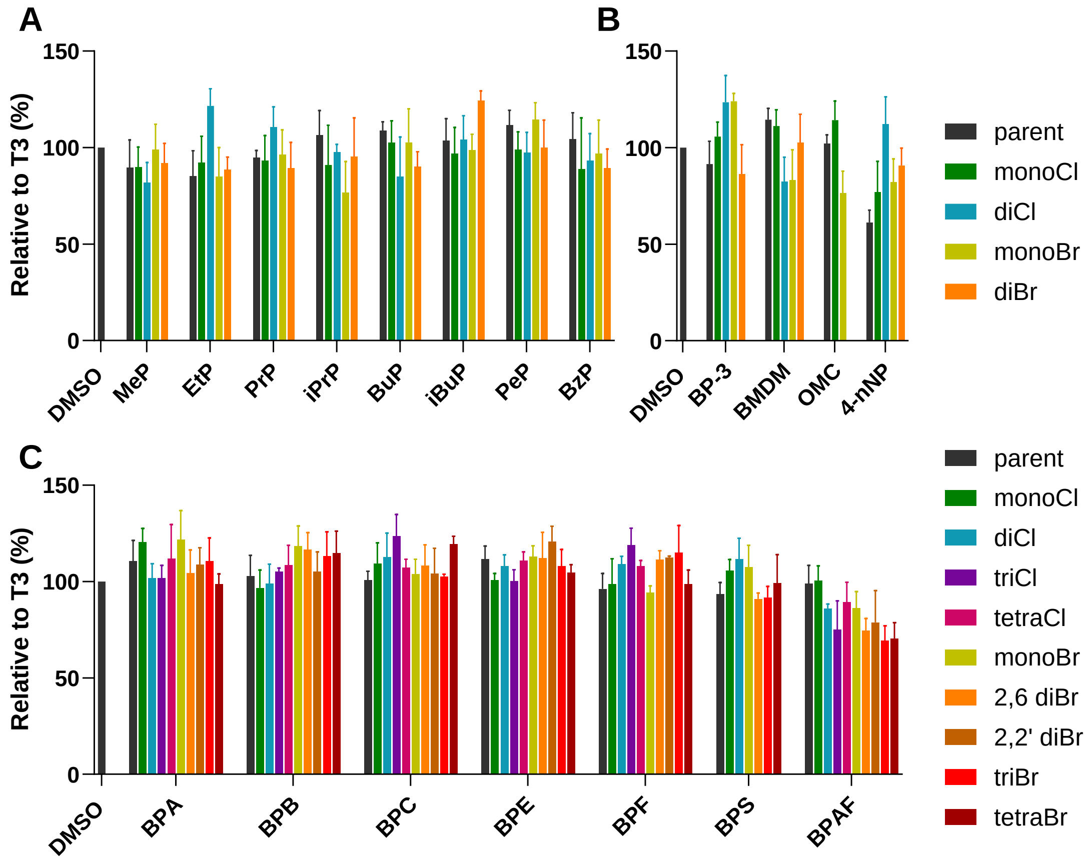

Figure S4. Relative thyroid receptor antagonistic activity of compounds at 1 µM concentration. GH3.TRE.Luc cells were treated with (A) parabens, (B) UV filters and nonylphenols and (C) bisphenols. After 1h, 0.25 nM of T3 was added to each well. DMSO was used as the negative control (0.1 %). After 24 h, luminescence was measured. Results are presented as mean ± SEM of four independent experiments. Statistical significance between tested compounds versus negative control (DMSO) was calculated using one-way ANOVA post hoc Dunnett's test. All the data was non-significant.

Supporting table

Table S1. Induction of GH3.TRE-Luc at the highest noncytotoxic concentration. All results are presented as fold induction ± SEM.

**Compound**	**Fold induction at 25 µM ± SEM**
BPF	1.73 ± 0.290
ClBPF	2.33 ± 0.375
Cl_2BPF	2.49 ± 0.568
Cl_3BPF	1.92 ± 0.337
Cl_4BPF	1.21 ± 0.184
BrBPF	2.72 ± 0.350
2,6-Br_2BPF	1.88 ± 0.196
2,2'-Br_2BPF	2.29 ± 0.480
Br_3BPF	1.10 ± 0.104
Br_4BPF	1.15 ± 0.0990
